# Uncontrollable voices and their relationship to gating deficits in schizophrenia

**DOI:** 10.1016/j.schres.2007.12.481

**Published:** 2008-04

**Authors:** Veena Kumari, Emmanuelle R. Peters, Dominic Fannon, Preethi Premkumar, Ingrid Aasen, Michael A. Cooke, Anantha P. Anilkumar, Elizabeth Kuipers

**Affiliations:** a Department of Psychology, Institute of Psychiatry, King's College London, London, UK; b Division of Psychological Medicine and Psychiatry, Institute of Psychiatry, King's College London, London, UK

**Keywords:** Auditory hallucinations, Anxiety, Prepulse inhibition, Startle, Schizophrenia

## Abstract

**Background:**

Prepulse inhibition (PPI) of the startle response refers to the ability of a weak prestimulus to transiently inhibit the response to a closely following strong sensory stimulus. This effect is reduced in a number of disorders known to be associated with impaired gating of sensory, cognitive or motor information. The aim of this study was to investigate PPI deficit in relation to the dimensions of auditory hallucinations in patients with schizophrenia or schizoaffective disorder.

**Method:**

PPI of the acoustically elicited eye blink startle response was measured electromyographically in 62 patients with schizophrenia (*n* = 55) or schizoaffective disorder (*n* = 7) (26 of 62 with current auditory hallucinations) and 22 healthy participants matched, on average, to age and sex of the patient group.

**Results:**

Patients, as a group, showed reduced PPI compared to healthy participants. The presence of auditory hallucinations was associated with a marked PPI deficit if the patients felt that they had no control over their occurrence and that they were unable to dismiss them. Hearing voices with a high degree of negative content was associated with high mean startle amplitude in patients with current auditory hallucinations.

**Conclusions:**

Although auditory hallucinations in patients with schizophrenia are theorised to result from impaired monitoring of inner speech, the inability to consciously ignore them appears to be associated with a gating deficit. Hearing voices with negative content is associated with hyper-startle responding, possibly because such voices are threatening and thus provoke anxiety.

## 1 Introduction

A key feature of schizophrenia is the inability to screen out irrelevant sensory input (Braff, 1993). Prepulse inhibition (PPI) of the startle response provides a valuable tool to study this feature (Geyer et al., 1990). PPI refers to a reliable reduction in the amplitude of the startle response to a strong sensory stimulus, the pulse, if this is preceded shortly (30–500 ms) by a weak stimulus, the prepulse (Graham, 1975).

PPI is found to be reduced in a number of neuropsychiatric disorders that are associated with impaired control of sensory (e.g. schizophrenia; Braff et al., 1978, review 2001; Kumari et al., 2005, 2007a,b; Meincke et al., 2004; Swerdlow et al., 2006), cognitive (e.g. obsessive compulsive disorder, Swerdlow et al., 1993; post-traumatic stress disorder, Ornitz and Pynoos, 1989; Grillon et al., 1996) or motor information (e.g. Huntington disease, Swerdlow et al., 1995). Within patients with schizophrenia PPI is reported to show weakly negative or no associations with positive or negative symptoms using ratings from general psychiatric symptom scales (review, Braff et al., 2001; Swerdlow et al., 2006) and relatively stronger negative correlations with thought disorder (Perry and Braff, 1994; Perry et al., 1999). PPI also correlates negatively with distractibility (Karper et al., 1996; Kumari et al., 2007b) and positively with functional status (Swerdlow et al., 2006) in schizophrenia.

Auditory hallucinations are common in schizophrenia and theorised to result from an impaired monitoring of inner speech (Frith, 1992). Auditory hallucinations are known to vary on a number of important dimensions other than frequency and severity, such as controllability and distress. Previous studies have noted associations between the frequency of auditory hallucinations and reduced suppression of irrelevant thoughts and memories (Waters et al., 2003; Badcock et al., 2005), and increased cognitive intrusions (Morrison and Baker, 2000). The inability to suppress intrusive memories and thoughts are considered to be the behavioural correlates of deficient PPI in patients with post-traumatic stress disorder (Grillon et al., 1996) and obsessive compulsive disorder (Swerdlow et al., 1993), respectively. No previous study to our knowledge has examined the influence of auditory hallucinations and its dimensions on PPI of the acoustic startle response in schizophrenia. Given earlier mentioned findings on the behavioural correlates of auditory hallucinations in schizophrenia (Grillon et al., 1996; Waters et al., 2003; Badcock et al., 2005) and the postulated link between PPI deficit and the inability to suppress intrusive memories and thoughts in patients with post-traumatic stress disorder (Grillon et al., 1996) and obsessive compulsive disorder (Swerdlow et al., 1993), perceived uncontrollability of voices may be associated with a marked PPI deficit in psychotic individuals.

In this study we examined PPI of the acoustic startle reflex in relation to the symptom of auditory hallucinations in patients with schizophrenia or schizoaffective disorder. Specifically, we tested the hypothesis that reduced controllability of voices would be associated with a more severe PPI deficit. The patient group, on average, was expected to show reduced PPI compared to the control group.

## 2 Materials and methods

### 2.1 Participants

The study sample included 62 patients with a diagnosis of schizophrenia (*n* = 55) or schizoaffective disorder (*n* = 7) who had agreed to take part in a longitudinal study to examine the biological effects of responsiveness to cognitive behaviour therapy (CBT) adjunct to their usual treatment (the data reported here were collected as part of their baseline assessments prior to any CBT) and 22 healthy participants. The healthy group was matched, on average, to age and sex of the patient group.

All patients were (i) recruited from the South London and Maudsley NHS Foundation Trust, (ii) on stable doses of antipsychotic medication for at least three months prior to taking part, (iii) in a chronic phase of the illness, and (iv) living in the community or long-stay/rehabilitation wards. Healthy participants were recruited from the local community.

Table 1 shows demographic and clinical characteristics of the study groups. The patients who reported hearing voices in the week preceding the clinical and psychophysiological assessments were classified as the group with current auditory hallucinations (AH+); remaining patients were classified as the group without current auditory hallucinations (AH−). The AH− group reported no auditory hallucinations when examined by the clinician and the researcher (in two separate sessions).

The study was approved by the South London and Maudsley NHS Foundation Trust and the Institute of Psychiatry research ethics committee. All participants provided written informed consent and were compensated for their time and travel.

### 2.2 Diagnoses and clinical assessments

Clinical diagnoses were made by an experienced psychiatrist (DF) using the Structured Clinical Interview for DSM-IV (First et al., 1995). Symptoms in patients were rated by the same psychiatrist using the Positive and Negative Syndrome scale (PANSS) (Kay et al., 1987). The patients with current auditory hallucinations were further rated using the auditory hallucinations subscale of the Psychiatric Symptom Ratings Scales (PSYRATS, Haddock et al., 1999) by a doctoral level psychologist (MC) who was not involved in psychophysiological data collection. The auditory hallucinations subscale of the PSYRATS scale has 11 items: frequency, duration, location, loudness, beliefs about the origin of voices, amount of negative content, degree of negative content, amount of distress, intensity of distress, disruption to life and controllability of voices. All items are rated on a five-point scale. The PSYRATS scales have shown excellent inter-rater reliability and validity in both chronic (Haddock et al., 1999) and first-episode schizophrenia samples (Drake et al., 2007). The positive symptom items of other clinical rating scales, namely, Scale for the Assessment of Positive Symptoms (SAPS; Andreasen, 1984) and PANSS (Kay et al., 1987), are reported to reflect more objective aspects of PSYRATS ratings of auditory hallucinations (frequency and duration items, *r* values > 0.30) but not the subjective aspects (*r* values between − 0.01 and 0.23 for location, loudness, amount of negative content, degree of negative content, amount of distress, intensity of distress, and controllability) (Steel et al., 2007).

All potential healthy participants were screened using a semi-structured interview (First et al., 2002) and included only if they did not have a history of mental illness, and drug and alcohol abuse and were not on a regular medical prescription.

In addition, predicted IQ was measured in all participants using the National Adults Reading test (Nelson and Willison, 1991) for sample characterization.

### 2.3 Psychophysiological data collection and scoring

A commercially available human startle response monitoring system (Mark II, SR-Lab, San Diego, California) was used to generate and deliver the acoustic stimuli, and to record and score the electromyographic (EMG) activity for 250 ms starting from the onset of the acoustic startle stimulus.

Acoustic stimuli were presented to participants binaurally through headphones. The pulse-alone stimulus was a 40-ms presentation of 114-dB (A) white noise and the prepulse stimulus a 20-ms presentation of 85-dB (A) white noise, both over 70-dB (A) continuous background noise. The noise levels were calibrated using the continuous noise, checked on a monthly basis, and re-calibrated if required. The session began with a 5 min acclimatization period consisting of 70 dB (A) continuous white noise. During the experiment, participants received four blocks of 12 trials each, after an initial pulse-alone trial; each block consisted of three pulse alone trials, three prepulse trials with a 30-ms prepulse-to-pulse (onset to onset) interval, three prepulse trials with a 60-ms prepulse-to-pulse interval, and three prepulse trials with a 120-ms prepulse-to-pulse interval presented to participants in a pseudorandom order with a mean inter-trial interval of 15 s (range 9–23 s).

The experimental procedures for recording and scoring the startle reflexes were identical to those reported previously (e.g. Kumari et al., 2000, 2005, 2007a). Eye blink component of the startle was indexed by recording EMG activity of the orbicularis oculi muscle directly beneath the right eye, by positioning two miniature silver/silver chloride electrodes. Recorded EMG activity was band-pass filtered, as recommended by the SR-Lab. A 50-hz filter was used to eliminate the 50-Hz interference. The EMG data were at first inspected on a trial-to-trial basis offline and then scored using the analytic programme of this system for response amplitude (in arbitrary analogue-to-digital units; one unit = 2.62 μV) and latencies to response onset and peak. Responses (< 4%) were rejected if the onset and peak latencies differed by more than 95 ms or when the baseline values shifted by more than 50 units. PPI was computed for each participant separately for each trial type as (*a* − *b* / *a*) × 100, where “*a*” = pulse-alone amplitude and “*b*” = amplitude over prepulse trials. Percent of PPI, rather than absolute amount of PPI (i.e. arithmetic difference between pulse-alone and prepulse trials), was used since this procedure eliminates the influence of individual differences in startle responsiveness. Psychophysiological data were scored blind to diagnosis and group membership.

Participants were told that the experiment was to measure their reaction to a number of noise-bursts, but no specific instructions were given on whether to attend or ignore them. They were requested to keep their eyes open during the experiment. There was no explicit restriction on smoking intake prior to testing but care was taken not to take participants to the startle laboratory for about 30 min after they had a cigarette, in order to prevent a state of smoking withdrawal or a heavy intake during the testing session that may transiently affect PPI (Kumari et al., 2001).

### 2.4 Data analysis

The demographic and clinical variables were analysed using one-way analysis of variance (ANOVA) with planned comparisons (LSD) to compare the three groups against one another, or using independent sample *t*-tests, as appropriate.

To examine possible group differences in the amplitude and habituation of the startle response over pulse-alone trials, the data were subjected to a 3 [Group: controls, AH+, patients, AH− patients] × 4 (Block: 4 blocks, each consisting of three trials) ANOVA, with Group as a between-subjects and Block as a within-subjects factor with planned comparisons. The possible associations of mean pulse-alone amplitude to pulse-alone trials over the entire session with age, duration of illness and PANSS symptom dimensions were examined across the entire sample using Pearson's correlations, and with dimensions of auditory hallucinations in the AH+ group using Spearman rank correlations. A significant correlation between hearing voices with a high degree of negative content and high mean startle amplitude in the AH+ group (see Results) was further examined by correlations between scores on individual items of the general psychopathology scale and mean pulse-alone amplitude using Spearman rank correlations. This enabled us to determine whether the observed associated may be mediated by heightened anxiety/tension (assessed with PANSS G2, anxiety, and G4, tension items) since anxiety states are well known to potentiate the startle response (review, Grillon, 2002).

To examine group differences in PPI, PPI (%) scores were subjected to a 3 (Group, as above) × 3 (Trial type: 30-ms, 60-ms and 120-ms prepulse trials) ANOVA, with Group as a between-subjects and Trial type as a within-subjects factor, with planned comparisons. The relationships of PPI scores to the PANSS symptom dimensions and other clinical and demographic variables were examined across the entire sample using Pearson's correlations. Possible associations between the dimensions of auditory hallucinations and PPI in the AH+ group were examined using Spearman rank correlations. Given that cigarette smoking (Kumari et al., 2001; George et al., 2006) and antipsychotic drugs (review, Kumari and Sharma, 2000; Leumann et al., 2002; Oranje et al., 2002; Kumari et al., 2002, 2007b; Swerdlow et al., 2006) influence PPI in schizophrenia, any association found between PPI and controllability dimension of auditory hallucinations was re-evaluated using partial correlations controlling for the number of cigarettes smoked per day (0 for non-smokers) and the type of antipsychotic medication (atypical, typical, both).

Latencies to response peak were analysed with a 3 (Group) × 4 (Trial Type: pulse-alone and 3 prepulse trials) ANOVA with Group as a between-subjects and Trial Type as a within-subjects factor. Following a significant Group effect in peak latency (see Results), this measure was further examined using Pearson's correlations in relation to clinical and demographic variables, and then using multiple regression models where more than one variable showed a significant association. The dimensions of auditory hallucination were also examined in association with the latency measure using the Spearman rank order correlations in the AH+ group. Following the observation of different clinical correlates of PPI and latency to response peak in our patient sample (see Results), we performed Pearson's correlations between PPI and latency to response peak at each prepulse type first in the entire patient sample, and then separately in the AH− and AH+ groups. We further evaluated any significant latency-PPI associations using partial correlations controlling for the duration of illness and PANSS symptom ratings and the controllability dimension of auditory hallucinations (AH+ group only).

All analyses were performed by SPSS windows (version 15). Alpha level for testing significance of effects was *p* = 0.05 unless stated otherwise. Sex was initially entered as a between-subjects factor in all ANOVAs but subsequently removed because it did not interact with Group in any analyses. Prior to conducting the described analysis procedures, the data were examined to ensure that statistical assumptions required for these analyses were met in this data set.

## 3 Results

### 3.1 Clinical and behavioural measures

As shown in Table 1, the three study groups did not differ in age (*F* < 1). Both AH+ and AH− patient groups, compared to the control group, had fewer years in education [*F*(2,84) = 3.16, *p* = 0.048; AH+ vs controls: *p* = 0.02; AH− vs controls; *p* = 0.03] and lower predicted IQ [*F*(2,84) = 7.16, *p* = 0.001; AH+ vs controls: *p* = 0.03 AH− vs controls; *p* = 0.001].

The AH+ and AH− groups were not significantly different in age at the onset of psychotic symptoms (as self-reported and verified with relatives and clinical records as much as possible) or the duration of illness (current age minus the age at onset of psychotic symptoms). The AH+ group, however, had more severe symptoms on the PANSS than the AH− group [positive symptoms: *t* (60) = 5.53, *p* < 0.001; negative symptoms: *t* = 2.56, *p* = 0.01; general psychopathology: *t* = 3.96, *p* < 0.001].

### 3.2 Startle measures

#### 3.2.1 Amplitude and habituation

The three groups showed comparable amplitude and habituation of the startle response over pulse-alone trials as demonstrated by a significant effect of Block [*F*(3, 243) = 25.159; *p* < 0.001) showing habituation of the startle response over four blocks (Linear *F*(1, 81) =36.22, *p* < 0.001) in all groups, but no Group or Group x Block effect (*F* < 1). Mean (s.e.m.) startle amplitudes for blocks 1–4 in the control and two patient groups are presented in Table 2.

Mean pulse-alone amplitude did not correlate significantly with age, duration of illness or PANSS symptom dimensions across the entire patient sample, but correlated positively with hearing voices with a high degree of negative content in the AH+ group (rho = 0.437, *p* = 0.04). Ratings on the PANSS G2 (anxiety: rho = 0.322, *p* = 0.008) and the PANSS G4 items (tension: rho = 0.278, *p* = 0.029) also correlated positively with mean pulse-alone amplitude across the whole sample (for all other general psychopathology items: *p* > 0.05) and (with somewhat larger rho values) when the analyses was restricted to the AH+ group only (PANSS G2: rho = 0.339, *p* = 0.09; PANSS G4: rho = 0.461, *p* = 0.018).

#### 3.2.2 PPI

**Fig. 1:**
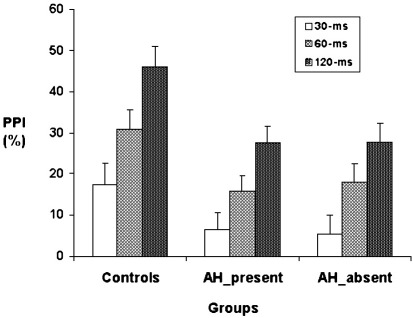
PPI (% inhibition) in groups of controls and patients with (AH-present) and without auditory hallucinations (AH-absent). Vertical lines demonstrate + 1 standard error of the mean.

There was, as expected, a significant effect of Group [*F*(2, 1) = 4.34, *p* = 0.016]. Planned comparisons showed less PPI in both patient groups relative to the control group (AH+ vs. controls: *p* = 0.01; AH− vs. controls; *p* = 0.01). The two patient groups were not significantly different from each other (Fig. 1). There was a significant effect of Trial type [*F*(2, 62) = 44.68; *p* < 0.001], reflecting a linear increase in PPI from 30-ms through 60-ms to 120-ms prepulse trials in all groups (Group × Trial type: *F* < 1).

**Fig. 2:**
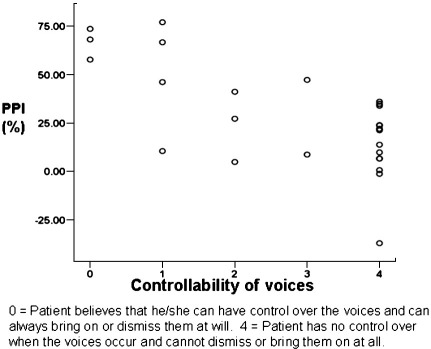
Association between control over hearing voices and PPI at 120-ms prepulse-to-pulse interval in patients with current auditory hallucinations.

Supporting our hypothesis, self-perceived lack of control over hearing voices was significantly associated with lower 120-ms (rho = 0.636, *p* < 0.001; Fig. 2) and 60-ms PPI (rho = 0.467, *p* = 0.02). This relationship was in the same direction for 30-ms PPI but failed to reach significance (rho = − 0.283, *p* = 0.16). The correlations between self-perceived lack of control over hearing voices and lower PPI were very similar and significant after we controlled for the number of cigarettes smoked daily (120-ms PPI: *r* = 0.689, *p* < 0.001; partial correlation = 0.671, *p* < 0.001; 60-ms PPI: *r* = 0.423, *p* = 0.03, partial correlation = 0.443, *p* = 0.027) and the type of antipsychotic treatment (120-ms PPI: partial correlation = 0.694, *p* < 0.001; 60-ms PPI: partial correlation = 0.403, *p* = 0.05). Demonstrating the specificity of controllability-PPI association, controllability dimension did not correlate (as mentioned earlier) with pulse-alone amplitude over the entire session, and no other dimension of auditory hallucinations correlated with PPI at any of three intervals (all *p* > 0.20).

No symptom dimension of the PANSS correlated with PPI across the entire sample (all *p* > 0.20). Age, predicted IQ, and number of years in education also did not significantly correlate with PPI at any of the three intervals (all *p* > 0.20).

#### 3.2.3 Peak latency

**Fig. 3:**
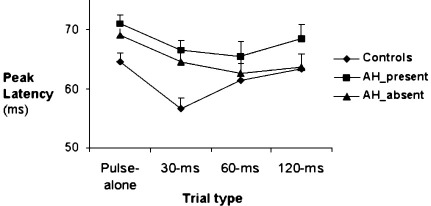
Peak latency in groups of controls and patients with and without auditory hallucinations. Vertical lines demonstrate + 1 standard error of the mean.

There was a significant main effect of Group [*F*(2, 81) = 3.78, *p* = 0.03] indicating significantly longer latencies in the AH+ group, relative to the control group, over all trial types (*p* = 0.007). Although the AH− patients did not differ significantly from controls, they also showed somewhat prolonged latencies (*p* = 0.11) and were not significantly different (*p* = 0.18) from the AH+ patients (Fig. 3). The main effect of Trial Type, as found in most previous PPI studies (reviewed, Braff et al., 2001; Kumari and Ettinger, 2005), was significant [*F*(3, 243) =8.78, *p* < 0.001] indicating latency facilitation by prepulse trials, especially with the 30-ms and 60-ms prepulse trials [linear *F*(1,81) = 4.06, *p* = 0.05; quadratic *F*(1,81) = 2680, *p* < 0.001] (Fig. 3). The Trial type × Group interaction was not significant (*p* = 0.17).

The results of correlational analyses (presented in Table 3) revealed that older age and a longer duration of illness most strongly correlated with longer latencies over various trial types. Negative symptoms and general psychopathology, but not positive symptoms, were also correlated with longer latencies over various prepulse trials (Table 3).

When examined in the entire patient sample, the multiple regression model for the latencies to pulse-alone amplitude (including age and the duration of illness) failed to reach formal significance [*F*(2,61) =2.78, *p* = 0.07; *R* = 0.29; *R*^2^ = 0.086]. The multiple regression model for latencies to 60-ms prepulse trials (including the duration of illness and the general psychopathology score) was significant [*F*(2,61) = 6.80, *p* = 0.002; *R* = 0.433; *R*^2^ = 0.188] with the duration of illness emerging as the only significant predictor (*t* = 2.96, *p* = 0.004). As shown in Table 3, only the duration of illness correlated significantly positively with latencies to the 30-ms and 120-ms prepulse trials (thus no multiple regression analyses).

When examined exclusively in the AH+ group, the multiple regression model for latencies to 30-ms prepulse trials (including the duration of illness, negative symptoms, general psychopathology) was significant [*F*(3,25) = 5.45, *p* = 0.006; *R* = 0.655; *R*^2^ = 0.428]; the duration of illness emerged as the only significant predictor (*t* = 2.64, *p* = 0.01). The multiple regression model for latencies to 120-ms prepulse trials (the duration of illness and age) was also significant [*F*(2,25) = 4.12, *p* = 0.03; *R* = 0.513; *R*^2^ = 0.264]; the duration of illness was the stronger of the two (age and the duration of illness) predictors. The dimensions of auditory hallucination did not show significant or consistent associations with the latency measure.

When examined across the entire patient sample, latencies to peak did not correlate significantly with PPI at 30-ms (*r* = − 0.189) or 60-ms intervals (*r* = − 0.072) but correlated significantly negatively with PPI at 120-ms intervals (*r* = − 0.257, *p* = 0.043). This effect was more marked and significant only in AH+ group when we examined these correlations separately in the AH− and AH+ groups. We found that latencies to peak did not correlate significantly with PPI at 30-ms (AH+ group: *r* = − 0.242; AH− group: *r* = − 0.169) or 60-ms intervals (AH+ group: *r* = − 0.158; AH− group: *r* = − 0.018) in either group, but correlated significantly negatively with PPI at 120-ms intervals in the AH+ group (AH+ group: *r* = − 0.436, *p* = 0.043; AH− group: *r* = − 0.138). The longer latencies-lower PPI association in the AH+ group remained significant after controlling for ratings on the controllability dimension of the PSYRATS (partial *r* = − 0.441, *p* = 0.027) and positive symptoms (partial *r* = − 0.439, *p* = 0.028), negative symptoms (partial *r* = − 0.437, *p* = 0.029) and general psychopathology scores on the PANSS (partial *r* = − 0.438, *p* = 0.029), but became non-significant after controlling for the duration of illness (partial *r* = − 0.30, *p* = 0.14).

Age was not significantly associated with latencies to pulse-alone or prepulse trials in the control group (all *p* > 0.35, *r* values from 0.07 to 0.20).

## 4 Discussion

Our results confirm a large number of previous reports of reduced PPI, on average, in patients with schizophrenia compared to healthy controls (Braff et al., 1978, review 2001; Kumari et al., 2000, 2005, 2007a,b; Meincke et al., 2004; Swerdlow et al., 2006). They also reveal, for the first time, that in schizophrenia patients (a) the presence of auditory hallucinations is associated with a deficit in PPI if the patients feel that they have no control over their occurrence, (b) hearing voices with a high degree of negative content is associated with hyper-startling, and (c) a longer duration of illness is more strongly associated than age with longer latencies to prepulse trials.

Of all neuropsychiatric disorders associated with impaired sensorimotor gating, PPI deficit has been most intensively studied in schizophrenia. As noted earlier in the Introduction, impaired PPI of the acoustic startle in this population is reported to be associated with thought disorder (Perry and Braff 1994; Perry et al., 1999) and distractibility (Karper et al., 1996), but found to be uncorrelated, or only weakly correlated, with positive symptoms (reviews, Braff et al., 2001 and Kumari and Ettinger, 2005; Swerdlow et al., 2006) assessed on the commonly used rating scales, namely, the PANSS (Kay et al., 1987) and the Scale for the Assessment of Positive Symptoms (Andreasen, 1984). The relationship between PPI and the subjective experiences of lack of control over hearing voices (i.e. the subjective awareness of cognitive disturbances rather than the principal feature of positive symptoms per se) may not be captured using the SAPS or the PANSS ratings of auditory hallucinations since they do not reflect the subjective aspects of auditory hallucinations (Steel et al., 2007).

Our results indicate that it is not the presence or absence of voices which is related to impaired PPI, but the feeling of having no control over their occurrence and being unable to dismiss them. This association may provide insight into possible mechanisms for recent observations of a positive relationship between PPI and functional status, but no significant association between PPI and positive or negative symptoms in the same sample (Swerdlow et al., 2006). It is conceivable that those with normal (to superior) sensorimotor gating are able to remain focussed on the task at hand and not be distracted by the voices, since they feel more able to exert control and dismiss them. While the presence of auditory hallucinations in patients with schizophrenia may well be explained by a self-monitoring and language-related deficit (Frith, 1992), our findings suggest that their perceived controllability is a function of their information processing characteristics, more specifically sensorimotor gating.

The observation that a deficit in PPI was present across both AH+ and AH− groups suggests that PPI deficit in schizophrenia is susceptible to multiple state- and trait-related influences (reviews, Braff et al., 2001; Kumari and Ettinger, 2005). It is highly unlikely that a single deficit or a specific symptom dimension will explain PPI deficit in the schizophrenia population as a whole.

Two other findings of our study also deserve some comment. First, hearing voices with a high degree of negative content was associated with a high pulse-alone amplitude in the AH+ group. This is not surprising given that such patients are likely to experience heightened anxiety and anxiety states are well known to potentiate the startle response (review, Grillon, 2002). The positive correlations found between the anxiety (G2) and tension (G4) items of the PANSS and high mean pulse-alone amplitude further suggest that the association between high pulse-alone amplitude and hearing voices with a high degree of negative content was mediated by anxiety and tension. Second, we found longer latencies to startle peak in the patient group. This effect was significantly and more markedly present in the AH+ group who also had more severe symptoms and a (non-significantly) longer duration of illness than the AH− group. The correlational analyses across all patients confirmed that this effect was associated with age (pulse-alone latency) and a longer duration of illness (latencies to prepulse trials). Although the severity of symptoms on the negative and general psychopathology dimensions of the PANSS also showed some association with latencies to prepulse trials, the duration of illness accounted for most of the total variance. We had not observed a schizophrenia-related deficit in the latency measure in previous studies from our laboratory (e.g. Kumari et al., 1999; 2000, 2002, 2005). The main reason for this might be that they included much smaller samples (*n* < 45) of less symptomatic, and mostly treatment-responsive, patients. A recent study by Swerdlow and colleagues (2006) involving a large cohort of patients (*n* = 103) has shown longer latencies in patients with schizophrenia; they found this effect to be age-related. Age in their healthy sample, as in ours, did not correlate significantly with latencies. The duration of illness seems to be a stronger predictor of longer latencies to prepulse trials than age in our patient sample. It is, however, difficult to disentangle the effect of duration of illness from that of ageing because the longitudinal effects of illness unfolded with age (a strong positive correlation between the two variables, *r* = 0.693; *p* < 0.001). The longer latencies-lower 120-ms PPI association found in the AH+ group was also, at least in part, mediated by the duration of illness. The possibility that patients with a longer duration of illness and in regular contact with psychiatric services over the longer term may have a more severe or different form of the illness, reflected for example here as ‘slowed processing of information’, deserves to be examined further in future prospective studies. The positive relationships that we found between longer latencies and negative and general psychopathology subscales of the PANSS also lend support to this possibility. It is perhaps interesting to note that PPI and latency measures had different clinical correlates in this study. These two measures are known to be differentially affected by manipulation of prepulse-to-pulse interval in healthy populations (greater latency facilitation at 30-ms prepulse-to-pulse interval but greater PPI at 120-ms prepulse-to-pulse interval) and considered to reflect different processes (Graham, 1975). We did not detect a schizophrenia-related deficit in habituation of the startle response over the experimental session. This was expected given that startle studies with the number of trials used in the current study do not find such a deficit (review, Braff et al., 2001). A much longer session with about 100 startle trials is generally required to detect such a deficit.

Finally, PPI is considered a well-validated animal model for evaluating existing and potential new drug treatments for schizophrenia (reviews, Swerdlow and Geyer, 1998; Geyer et al., 2001). There is considerable evidence that effective medication, more so with atypical antipsychotics, can at least partially correct PPI deficit in schizophrenia patients (Kumari et al., 1999, 2000, 2002, 2007b; Weike et al., 2000; Leumann et al., 2002; Meincke et al., 2004; Swerdlow et al., 2006). The patients in the present study were medicated with antipsychotic drugs (including clozapine) most suited to them but still, as a group, showed robust deficit at all prepulse intervals. The most likely reason for this is that over 70% of our patients were considered to have shown insufficient clinical improvement by their treating clinicians and wished to receive cognitive behaviour therapy in addition to their usual drug treatment. Our findings thus raise the interesting possibility that a lack of (or reduced) improvement in PPI may help to characterize resistance to pharmacotherapy in schizophrenia.

With replication, the present findings may have theoretical and practical implications. At the theoretical level, our findings suggest that subjective perceptions of psychotic symptoms have specific neurophysiological, and possibly functional outcome, correlates. Such associations are unlikely to be detected with commonly used (clinician-oriented and unidimensional) symptom rating scales. At the clinical level, appraisals of control over voices may be difficult to change since they seem to be related to an underlying gating deficit.

### 4.1 Limitations

Our study suffers from two limitations. Firstly, the majority of study participants, as is often the case in studies of schizophrenia patients, were male. Secondly, a large proportion of patients had remained distressed by one or more of their symptoms despite stable medication (and having tried several antipsychotics in the past) and wished to receive cognitive behaviour therapy in addition to their usual drug treatment. The findings thus may not generalise to the schizophrenia population on the whole.

## 5 Conclusions

In conclusion, this study found that the presence of auditory hallucinations is associated with a deficit in PPI if the patients feel that they have no control over their occurrence. This finding can be taken to suggest that subjective perceptions of psychotic symptoms have specific neurophysiological, and possibly functional outcome, correlates. It also found that hearing voices with a high degree of negative content is associated with hyper-startle responding possibly because such voices provoke anxiety and tension in patients with schizophrenia.

## Role of the funding source

The sponsor (the Wellcome Trust) had no role in study design; in the collection, analysis and interpretation of data; in the writing of the report; or in the decision to submit the paper for publication.

## Contributors

VK, EP, and EK designed the study. DF and AA performed the diagnostic interviews and clinical assessments. PP, IA and MC collected the data. VK undertook the statistical analysis and prepared the first draft. All authors contributed to and approved the final manuscript.

## Conflict of interest

The authors declare no conflict of interest.

## Figures and Tables

**Table 1:** Demographics and clinical characteristics of study groups

	Healthy participants (*n* = 22)	Patients with current auditory hallucinations (*n* = 26)	Patients with previous or no hallucinations (*n* = 36)
	Mean (s.d.)	Mean (s.d.)	Mean (s.d.)
*Demographics*			
Age (years)	35.23 (12.72)	38.73 (9.08)	38.81 (10.70)
Predicted IQ (aNART)	115.23 (8.47)	109.81 (9.13)	105.37 (8.47)
Years of education	15.18 (2.75)	13.46 (2.45)	13.68 (2.59)
Sex distribution (male/female)	16/6	18/8	28/8
Smokers (*n*)	4	12	22
< 10 cigarettes/daily	1	2	7
10–20 cigarettes/daily	2	7	7
> 21 cigarettes/daily	1	3	8

*Clinical Characteristics*			
Diagnoses		25 schizophrenia (24 paranoid, 1 catatonic), 1 schizoaffective disorder	30 schizophrenia (27 paranoid, 3 residual), 6 schizoaffective disorder
Age at onset of psychotic symptoms (years)		21.62 (6.56)	25.14 (8.63)
Duration of illness (current age minus age at onset of symptoms)		17.12 (10.06)	13.67 (10.41)
bPositive Symptoms		19.92 (3.73)	14.28 (4.12)
bNegative Symptoms		20.11 (3.97)	17.22 (4.67)
bGeneral Psychopathology		35.46 (6.98)	31.53 (6.02)
Total b PANSS Scores		75.50 (11.71)	63.03 (12.62)
Medication (*n*)		19 patients on atypical antipsychotics, 2 patients on typical antipsychotics, 4 patients on both atypical and typical antipsychotics and 1 patient medication non-compliant.	28 patients on atypical antipsychotics, 7 patients on typical antipsychotics, 1 patient on both atypical and typical antipsychotics.

a NART: National Adult Reading Test (Nelson and Willison, 1991).

b Positive and Negative Syndrome Scale (PANSS; Kay et al., 1987).

**Table 2:** Mean (standard error of the mean, s.e.m.) response amplitudes (in analogue-to-digit units; 1 unit = 2.62 μV) over the four blocks of three pulse-alone trials each and latencies to response onset in patients with schizophrenia and controls

	Healthy participants	Patients with current auditory hallucinations	Patients with previous or no hallucinations
Startle amplitude	Mean (s.e.m.)	Mean (s.e.m.)	Mean (s.e.m.)

Block 1	256.18 (44.87)	254.49 (41.28)	239.82 (35.08)
Block 2	225.05 (33.90)	176.94 (31.18)	180.88 (26.50)
Block 3	188.30 (33.20)	154.55 (30.54)	161.80 (25.95)
Block 4	176.80 (27.51)	131.01 (25.31)	134.11 (21.51)

**Table 3:** Correlations (Pearson's *r*) between peak latency and demographic and clinical variables in patients with current auditory hallucinations and for the entire patient sample (in parentheses)

	Pulse-alone	30-ms	60-ms	120-ms
Years of education	− 0.050 (0.151)	0.029 (0.023)	− 0.060 (− 0.046)	0.171 (− 0.025)
Predicted IQ (aNART)	− 0.093 (− 0.069)	− 0.144 (− 0.151)	− 0.059 (0.093)	0.0176 (0.134)
Age	0.345 (**0.272⁎**)	0.325 (0.211)	0.289 (0.195)	0.398^⁎^ (0.100)
Age at onset of psychotic symptoms	0.147 (0.008)	− 0.219 (0.116)	− 0.076 (− 0.240)	− 0.238 (0.239)
Duration of illness	0.215 (**0.269⁎**)	**0.438⁎ (0.292⁎)**	0.311 (**0.373⁎⁎**)	**0.513⁎⁎(0.280⁎⁎)**
bPositive symptoms	− 0.149 (− 0.022)	0.015 (0.030)	0.082 (0.136)	0.056 (0.124)
bNegative symptoms	0.373 (0.190)	**0.389⁎** (0.187)	0.335 (0.206)	0.068 (0.238)
bGeneral psychopathology	0.179 (0.140)	**0.430⁎** (0.237)	0.254 (**0.259⁎**)	0.034 (0.184)
Total bPANSS scores	0.186 (0.124)	**0.424⁎** (0.189)	0.291 (0.244)	0.062 (0.214)

^⁎^*p* < 0.05, ^⁎⁎^*p* < 0.01.

a NART: National Adult Reading Test (Nelson and Willison, 1991).

b Positive and Negative Syndrome Scale (PANSS; Kay et al., 1987).
